# Dermato-Neuro Syndrome After Intravenous Immunoglobulin Infusion: Case Report

**DOI:** 10.3390/reports9020148

**Published:** 2026-05-12

**Authors:** Bryce Kassalow, Soha Kazmi, Said Shukri, Zachary N. London

**Affiliations:** 1 Medical School, University of Michigan, Ann Arbor, MI 48109, USA; 2 Department of Neurology, University of Michigan, Ann Arbor, MI 48109, USAzlondon@med.umich.edu (Z.N.L.)

**Keywords:** dermato-neuro syndrome, neuroimmunology, intravenous immunoglobulin, hyperviscosity, hyperCKemia

## Abstract

**Background and Clinical Significance**: Dermato-neuro syndrome is a rare, potentially fatal complication of scleromyxedema, characterized by a prodrome of flu-like symptoms, and a triad of fever, confusion, and seizures. Intravenous immunoglobulin (IVIG) has become first-line treatment for both scleromyxedema and dermato-neuro syndrome based on case reports and case series data showing variable treatment responses. **Case Presentation**: In this report, we describe a Black, female patient with scleromyxedema and lambda-restricted IgG monoclonal gammopathy who developed suspected dermato-neuro syndrome within a week of her first round of IVIG infusions. **Conclusions**: To our knowledge, this is the second case report of dermato-neuro syndrome temporally linked to a recent IVIG infusion, a paradoxical reaction that may complicate clinical decision making. Furthermore, we highlight the dermatologic manifestations of scleromyxedema in dark skin tones and emphasize the need for heightened clinical suspicion of dermato-neuro syndrome in patients with scleromyxedema presenting with acute neurological symptoms.

## 1. Introduction and Clinical Significance

Scleromyxedema is a rare systemic disorder characterized by fibroblast dysregulation and excessive mucin deposition, and is associated with significant mortality and morbidity [1,2,3]. The disease generally affects middle aged adults (30–80 years of age) with no sex or racial predominance. As of 2022, there were fewer than 200 reported cases of scleromyxedema in the literature [4]. The diagnosis for scleromyxedema requires: (1) generalized papular and sclerodermoid eruption; (2) histological evidence of mucin deposition, fibroblast proliferation, and fibrosis; (3) monoclonal gammopathy (most often IgG λ); and (4) the absence of thyroid disease [2]. The generalized nature of the papular eruption distinguishes it from lichen myxedematosus, a more common, focal cutaneous mucinosis, and the absence of thyroid disease distinguishes scleromyxedema from myxedema secondary to excessive glycosaminoglycan production in the setting of severe thyroid dysfunction. Though researchers have been studying this disease for over forty years, the pathogenesis of scleromyxedema is poorly understood. The prevailing hypothesis is that circulating cytokines, such as IL-6, TNFα, and TGFβ, stimulate glycosaminoglycan synthesis, fibroblast proliferation, and mucin deposition; however, a characteristic abnormality in fibroblasts has not yet been identified and the quantity of mucin deposits does not correlate with disease severity [5]. The role of the associated, concurrent monoclonal gammopathy in the disease process is also not understood, as paraprotein levels do not appear to correlate with the severity or progression of the disease [5].

Clinically, scleromyxedema typically presents with small (<1 cm), waxy, firm papules on the face, neck, chest, back, and arms before progressing to systemic involvement and organ dysfunction over the course of years. In existing case series, the mean time from primary symptoms to diagnosis was 10–41.6 months highlighting the diagnostic challenge of spotting this rare disease [1]. The characteristic, flat-topped, waxy, and raised papules of scleromyxedema are frequently misdiagnosed as acne. Without treatment, the disease usually progresses over the course of years to affect multiple organ systems. The most common extracutaneous manifestations are neurologic (30% of patients), rheumatologic (23.3% of patients), and cardiac (20% of patients) including but not limited to dermato-neuro syndrome, rhabdomyolysis, proximal muscle weakness, carpal tunnel syndrome, and mucinous cardiopathy [1].

Dermato-neuro syndrome, a particularly severe complication of scleromyxedema, was initially defined by the diagnostic triad of fever, seizures, and coma [6]. In the three case series conducted on scleromyxedema, 4% (*n* = 30) [1], 18% (*n* = 33) [7], and 32% (*n* = 19) [8] of patients were noted to suffer one or more episodes of dermato-neuro syndrome. Multiple deaths have also been associated with dermato-neuro syndrome episodes in these case series. Though dermato-neuro syndrome was initially defined by the diagnostic triad of fever, seizures, and coma, recent reports have since suggested broadening the definition to encompass any acute, unexplained neurological deficit in scleromyxedema patients, reflecting an increasing recognition of its phenotypic heterogeneity [9].

The pathophysiology of dermato-neuro syndrome remains poorly understood. No unique laboratory findings have been identified to aid in diagnosis, neuroimaging studies are typically unremarkable, and there are no randomized clinical trials to inform treatment [1,8]. Given the rarity and nonspecific presentation of dermato-neuro syndrome, clinicians depend on anecdotal evidence from case reports and case series for diagnostic and therapeutic guidance. While IVIG has emerged as a first-line treatment for non-severe, skin-limited scleromyxedema [7], its utility in severe cases, such as those that progress to dermato-neuro syndrome, remains unknown [10].

In this paper, we present the case of a 49-year-old Black woman diagnosed with scleromyxedema, on whom neurology was consulted to work up the etiology of her acutely altered mental status 4 days after an initial infusion of IVIG (0.5 mg/kg/day for four days). Our case report explores: (1) the paradoxical role of IVIG as both a treatment for scleromyxedema and potential trigger of dermato-neuro syndrome in this patient; (2) the need for heightened clinical suspicion in scleromyxedema patients presenting with altered mental status; and (3) the dermatological manifestations in skin of color, and their potential to delay diagnosis.

## 2. Case Presentation

**Figure 1:**
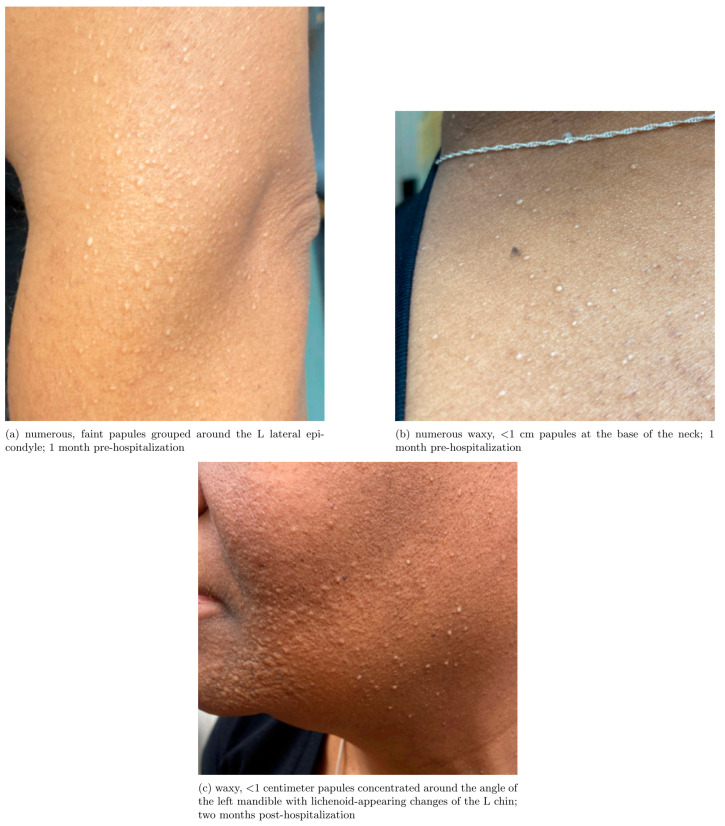
Relevant skin findings around the time of hospitalization. Waxy papules on the (**a**) left arm, (**b**) neck, and (**c**) left cheek.

A 49-year-old Black female with a past medical history notable for IgGλ restricted smoldering myeloma and scleromyxedema presented to the ED with fatigue, nausea, vomiting, loss of appetite, and pain in her shoulders and calves which began two days after her first IVIG infusion for scleromyxedema. She initially presented to dermatology nine months prior to this hospitalization for a 7+ year history of waxy papules on her face, grouped around and under her mandible bilaterally and superior neck. These papules were previously diagnosed as acne and were treated unsuccessfully with a topical steroid treatment (triamcinolone 0.1% cream). After a year of unsuccessful treatment for suspected acne she noticed an increased distribution of the papular rash, which included the posterolateral aspect of her bilateral arms (particularly around her lateral epicondyles bilaterally), her lower neck and upper sternum, and her bilateral anterior legs (Figure 1). A skin biopsy of the left central buccal cheek was performed and the report from an outside provider described that formalin-preserved sections showed signs of fibroblast proliferation with colloidal iron stain confirming increased dermal mucin deposition, consistent with papular mucinosis. Further workup at the time included a protein electrophoresis which showed an IgGλ restricted smoldering myeloma, which in conjunction with her skin findings led to a diagnosis of scleromyxedema. In the six months leading up to hospitalization, lesions of her left lower face worsened with dense collections of papules coalescing into plaque forming along the angle of her left mandible. Her cutaneous lesions also continued to spread from her chin to her neck, chest, and arms, prompting initiation of IVIG therapy, which was the first treatment she received for her scleromyxedema. She did not receive any treatment for her smoldering myeloma or any other initial treatment for her scleromyxedema prior to hospitalization.

On presentation to the emergency department, she was afebrile, with a blood pressure elevated from baseline (165/96), tachycardia up to 116 beats per minute, and tachypnea up to 25 breaths per minute. Initially, the patient was conversant and appropriate, but over her first 24 h in the hospital, she became obtunded, disoriented to person, place, and time, and arousable only to painful stimuli. She was admitted to a general medicine floor and neurology was consulted to explore possible etiologies of her acutely altered mental status and fatigue.

Her initial workup was notable for a normal brain MRI, rapidly rising creatine phosphokinase (CPK) and white blood cell (WBC) count (Table 1 and Table 2). Though some case reports support the use of IVIG for management of dermato-neuro syndrome, this patient was treated supportively because of concerns about the temporal relationship between her first IVIG infusion and symptom onset. Her mental status, confusion, WBC, and CPK slowly began improving on day 5 of hospitalization. At the time of discharge, 14 days after admission, she was able to sit up in a chair and communicate slowly with staff.

After discharge, she reinitiated monthly IVIG infusions for her scleromyxedema without adverse effects. Neuropsychiatric testing three months post-discharge was notable for cognitive testing within normal limits but nine months post-hospitalization, the patient continued to report that her cognitive function had not returned to its prior baseline. 14 months after hospitalization, she continues to receive regular IVIG infusions, which she restarted the month after hospitalization, and follows up regularly with a hematologist, rheumatologist, and neurologist. She reports that the number of skin lesions on her body has notably decreased, which is validated by images available in the chart. The patient explicitly consented to the writing of this case report and signed a consent form allowing for the publication of her de-identified health data.

There are several features suggesting that her acute presentation was due to dermato-neuro syndrome, including her history of IgG lambda restricted smoldering myeloma and scleromyxedema, acutely altered mental status with a flu-like prodrome, EEG consistent with moderate encephalopathy, rapidly rising creatine phosphokinase, aseptic lymphocytic pleocytosis of the cerebrospinal fluid, and lack of alternative diagnoses to explain these findings.

Though the patient’s presentation is consistent with dermato-neuro syndrome, a broad differential was explored. Alternative diagnoses that were considered included but were not limited to bacterial, viral, and fungal meningitis, dermatomyositis, statin-induced myopathy, paraneoplastic process, leptomeningeal carcinomatosis, sarcoidosis, systemic lupus erythematosus, and AL amyloidosis. IVIG-induced aseptic meningitis was seriously considered as an alternative diagnosis, but would not explain this patient’s altered mental status and would typically present with headache, nuchal rigidity, and photophobia, none of which were noted. A viral encephalitis could explain her altered mental status and WBC trend (Table 1), but extensive viral testing of the cerebrospinal fluid was negative (Table 2), brain MRI was unremarkable, and there were no focal abnormalities on EEG.

## 3. Discussion

Dermato-neuro syndrome remains a serious and poorly understood complication of scleromyxedema. Given the litany of negative test results (Table 1 and Table 2), we arrived at a diagnosis of dermato-neuro syndrome by exclusion after investigating alternative etiologies. However, we acknowledge that the absence of a definitive confirmatory test means that alternative diagnoses cannot be ruled out. This diagnostic uncertainty reflects a broader challenge in the field as the diagnostic criteria for dermato-neuro syndrome remains a subject of active debate.

Researchers have cautioned against both the overdiagnosis and underdiagnosis of dermato-neuro syndrome given the nonspecific clinical presentation. One recent paper warned that dermato-neuro syndrome may be overdiagnosed due to overlapping features with infection-induced acute encephalitis [11], while others have argued that the diagnostic criteria should be broadened to capture atypical presentations and reduce the risk of missed diagnoses [9]. One paper proposed expanding the definition of dermato-neuro syndrome to include any unexplained neurological deficit in scleromyxedema patients, demonstrating the importance of clinical judgment in ambiguous cases [9].

One prominent pathophysiological theory of dermato-neuro syndrome in the literature posits that IL-6 overactivation elicits a hyperpermeable blood–brain barrier leading to paraprotein-induced cerebrovascular sludging and encephalopathic mental status changes [6,12]. Elevated IL-6 levels in cerebrospinal fluid have been documented in two dermato-neuro syndrome patients [8,13]. This proposed mechanism implicates hyperproteinemia and paraproteinemia in increasing blood viscosity and impairing microcirculation, potentially contributing to encephalopathy. Building on this theory, emerging reports question whether infections and immunologic stressors (e.g., viral infection, IVIG) may precipitate dermato-neuro syndrome via a similar, IL-6 mediated mechanism, though this theory, of course, also relies upon limited data from case reports [13,14].

The chronology of our patient’s disease course suggests the possibility that IVIG may have contributed to the development of dermato-neuro syndrome. This is the second case in the literature highlighting a temporal association between IVIG and dermato-neuro syndrome [15], raising the question of whether IVIG may be acting as an immunologic stressor in some scleromyxedema patients. In the prior case report suggesting a temporal relationship between IVIG infusion and dermato-neuro syndrome, the patient similarly experienced symptoms of fever, malaise, and myalgia within two days of IVIG infusion followed by incoherent speech and altered mental status four days later. Notably, this report described a patient who already experienced a prior episode of dermato-neuro syndrome 15 years prior as well as numerous IVIG infusion treatments; therefore, this would be the first report of a patient experiencing an episode of dermato-neuro syndrome after her initial IVIG infusion [15].

**Figure 2:**
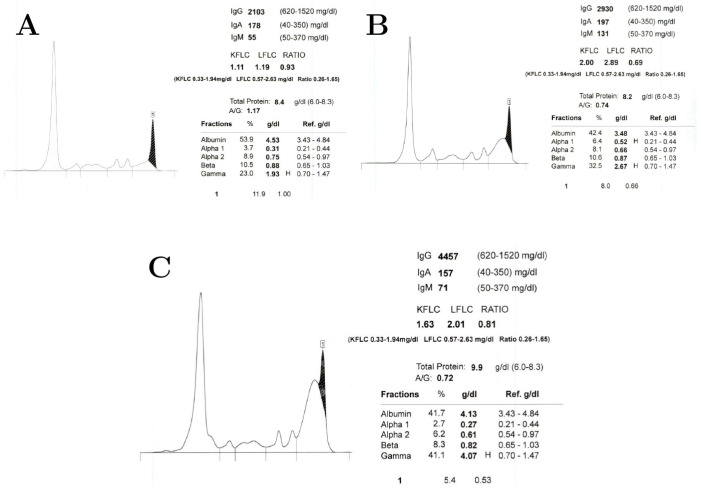
Serial protein electrophoresis results. (**A**) Electrophoresis results 3 months prior to initiation of IVIG and hospitalization. (**B**) Electrophoresis results during hospitalization, 14 days after initial IVIG infusion. (**C**) Electrophoresis 8 months after hospitalization and continued IVIG therapy. Filled-in black area under the curve represents the monoclonal M-spike within the IgG range. “H” stands for “high”, representing a value above a test’s respective refernece range.

The proposed mechanisms of IVIG include the neutralization of pathogenic antibodies, clearance of immune complexes, blocking Fc receptors, and modulating immune response and cytokines [5,10]. It is worth considering whether IVIG may contribute to a proposed cerebrovascular sludging effect via its modulation of cytokines, its disputed upregulation of IL-6 [16,17], and/or through its well-documented transient elevation of serum protein concentration [18]. Serial protein electrophoresis results from three months prior to our patient’s hospitalization, during hospitalization, and 8 months after hospitalization (Figure 2) highlight that the majority of this patient’s serum protein elevation at the time of hospitalization can be attributed to an elevated polyclonal IgG population, likely secondary to IVIG therapy.

However, hyperviscosity or hyperproteinemia alone cannot explain this patient’s presentation. This patient’s IgG level at the time of hospitalization (~2930 mg/dL), while elevated (reference range 600–1600 mg/dL), fell short of the 10,000 mg/dL threshold that is typically expected in IgG-induced hyperviscosity syndrome in otherwise healthy patients [19,20]. Furthermore, the patient was reinitiated on IVIG therapy after hospitalization, and her most recent protein electrophoresis results (Figure 2C), 8 months post-hospitalization, demonstrated an even higher IgG level (4457 mg/dL) and total protein (9.9 g/dL) than at time of hospitalization without any recurrence of dermato-neuro syndrome symptoms. For these reasons, this case of dermato-neuro syndrome cannot be singularly explained by IVIG-induced hyperviscosity or hyperproteinemia.

Unfortunately, there are no definitive guidelines for the treatments of scleromyxedema or dermato-neuro syndrome as the rarity of these conditions hinders the feasibility of randomized clinical trials to evaluate different treatment modalities and clinical evidence relies on observational data. IVIG is the most commonly prescribed treatment for scleromyxedema with high rates of skin (69%), systemic (63%), and paraproteinemia responses (75%). Second-line treatments include corticosteroids and thalidomine (either alone or in combination with IVIG), and third-line treatments include autologous bone marrow transplant, melphalan, and bortezomib with dexamethasone [21].

Treatment data for dermato-neuro syndrome are even scarcer than for scleromyxedema, though case reports have described successful treatment with IVIG, plasmapheresis, corticosteroids, and in some cases supportive care. Plasmapheresis may directly reduce proteinemia and the success of plasmapheresis treatment lends credence to the hyperviscosity hypothesis. In one case report, plasmapheresis was successful in treating a patient unresponsive to IVIG treatment. Paradoxically, some scleromyxedema treatments may trigger dermato-neuro syndrome; there has been one case published report of autologous stem cell transplantation for scleromyxedema triggering dermato-neuro syndrome, while two reports now draw a temporal link between IVIG infusion and dermato-neuro syndrome [10,13,15].

Lastly, initial skin changes in scleromyxedema can easily be missed or misdiagnosed by physicians as acne or other dermatologic mimics. With the awareness that patients with dark skin are underrepresented in medical education resources [22] and that this disparity may contribute to reduced diagnostic accuracy and confidence of dermatologic conditions in patients with dark skin [23], we have shared additional, enhanced images of our patient’s skin findings in Appendix A. Upon our review, one common phrase used to describe scleromyxedema skin findings in the literature is “flesh-colored”; however, this patient’s skin findings show a grouped, papular, white rash that for this patient of color does not appear “flesh colored,” highlighting the limitations of broadly applying that description to mucinous skin lesions. In her case, these flat-topped, white lesions were mistaken for comedonal acne, a common misdiagnosis of cutaneous mucinous skin lesions [1]. Poor response to topical treatment and worsening spread of the patient’s rash prompted her dermatologist to perform a skin biopsy. Additional patient history that may increase suspicion for a systemic mucinosis includes, but is not limited to, worsening fatigue, progressive dyspnea, progressive dysphagia, progressive compressive neuropathy, episodes of acutely altered mental status, or new pitting edema of the lower extremities. This case highlights that symptoms of systemic mucinoses are often non-specific. However, in a patient with acute, unexplained altered mental status, a thorough dermatologic examination and history can reveal important diagnostic clues that help guide clinicians to the correct diagnosis.

As with any case report, our findings have inherent limitations. The experience of a single patient limits generalizability, and incomplete data and confounding variables are unavoidable. In this case, diagnostic uncertainty persists, and a causal relationship between IVIG administration and the onset of dermato-neuro syndrome cannot be established from one case alone. The possibility that this case represents IVIG-refractory dermato-neuro syndrome remains possible and it is important to clarify that the temporal connection between this patient’s IVIG infusion and her symptom onset is explored in this paper as a hypothesis-generating exercise rather than a suggestion of causality.

## 4. Conclusions

Dermato-neuro syndrome is a rare but deadly complication of scleromyxedema that can present as acutely altered mental status and a markedly elevated CPK. It would serve neurologists to keep dermato-neuro syndrome in mind when consulted on patients with acutely altered mental status or elevated CPK in the setting of known paraproteinemia and either scleromyxedema or unusual skin findings. This case report raises the possibility of IVIG contributing to the onset of dermato-neuro syndrome in scleromyxedema. Further studies and similar reports may help elucidate the pathophysiology of dermato-neuro syndrome and how IVIG impacts both its pathogenesis and treatment. Given the limitations of studying rare diseases, physicians should balance the reported benefits of IVIG treatment in scleromyxedema patients with its potential risks.

## Figures and Tables

**Table 1:** Laboratory and diagnostic findings across hospitalization. Day 0 represents day of presentation to the emergency department.

Test (Unit; Reference Range)	Day 0	Day 1	Day 2	Day 3	Day 4	Day 5	Day 6	Day 7	Day 8	Day 9	Day 10
WBC (×10^9^/L, 4.0–11.0	13.0	17.0	16.8	18.8	23.8	28	27.5	18.8	12.7	11.2	9.7
Hgb (×10^9^/L; 12.0–16.0)	11.1	8.8	9.0	8.7	9.3	8.5	7.4	8.7	8.4	8.0	8.2
Creatine phosphokinase (U/L; 26–180)	682	2596	6789	9213	12,516	9105	4610	1870	1266	464	256
AST (U/L; <34)	37	92	110	191	247	211	146	88	82	49	36
ALT (U/L; 10–49)	16	22	46	65	76	72	55	61	48	38	31
Serum Protein (g/dL; 6.0–8.3)	9.4	8.5	8.5	8.5	8.3	8.2	7.4	8.4	7.6	7.2	7.3
Sodium (mmol/L; 136–146)	130	132	130	131	133	134	138	135	138	137	136
Glucose (mg/dL; 70–180)	181	132	185	166	133	121	96	118	123	123	132
ESR (mm/h; 0–20)	73	40	62	56	64	62	49	88	69	87	-
CRP (mg/L; <0.6)	1.8	2.4	3.3	2.5	1.5	1.4	1.6	2.6	3.0	2.4	2.3
Whole Blood Viscosity (cP)	-	-	-	47.0	-	-	-	-	-	-	-
Plasma Viscosity (cP; 1.35–1.85)	-	-	-	2.19	-	-	-	-	-	-	-
LDH (U/L; 120–240)	-	655	-	-	-	-	-	-	-	-	-
Myoglobin, urine (ng/mL; <45)	-	1340	-	-	-	-	-	-	-	-	-
Thyroid Stimulating Hormone (mIU/L; 0.3–5.5)	0.89	-	-	-	-	-	-	-	-	-	-

**Table 2:** Further pathology, radiology, microbiology, electroencephalography, and laboratory workup.

Test (Unit; Reference Range)	Result	Day of Hospitalization Collected
Microbiology work-up of plasma and urine	Hepatitis C RNA by PCR not detected, Epstein–Barr virus DNA by PCR not detected, cytomegalovirus DNA by PCR not detected, aerobic and anaerobic bacterial cultures showed no growth, acid-fast bacilli culture showed no growth, fungal culture showed no growth	Day 1
Myomarker3 Panel (CU, <20)	Negative Anti-Mi2-Ab, <20 Anti-Jo-1 antibody, <20 Anti-Tif1-gamma antibody	Day 2
HMG-CoA reductase antibody (CU, <20)	<20	Day 2
MR Femur	No evidence of myositis involving the thighs	Day 2
MR Brain	No evidence of acute process	Day 3
EEG	Background activity was marked by diffuse delta admixed theta frequencies. There was no posterior dominant rhythm, and the AP gradient was not well-formed. During behavioral sleep, there were no sleep architectures. Activation was not performed. There were no epileptiform discharges, focal abnormalities, or seizures recorded during the EEG	Day 3
Cerebrospinal Fluid Studies	Lymphocytic pleocytosis (100 cells counted, 76 lymphocytes), protein 82, glucose 79, red blood cell count 1	Day 4
Flow cytometry and immunophenotyping of cerebrospinal fluid	Paucicellular specimen with no immunophenotypic evidence of monoclonal B cells or aberrant T cells. B-cell Kappa/lambda ratio: 2.43	Day 4
Microbiology work-up of cerebrospinal fluid	Human herpes virus 8 DNA by PCR not detected, herpes simplex virus-1 and herpes simplex virus-2 DNA by PCR not detected, varicella DNA by PCR not detected, cryptococcal antigen not detected, syphilis VDRL not detected, bacterial culture yielded no growth, acid-fast bacilli culture yielded no growth, fungal culture yielded no growth	Day 4
Chemokine Ligand 9 (pg/mL, <647)	2145	Day 5
Muscle Biopsy	Diffuse type 2 fiber atrophy	Day 8

**Figure A1:**
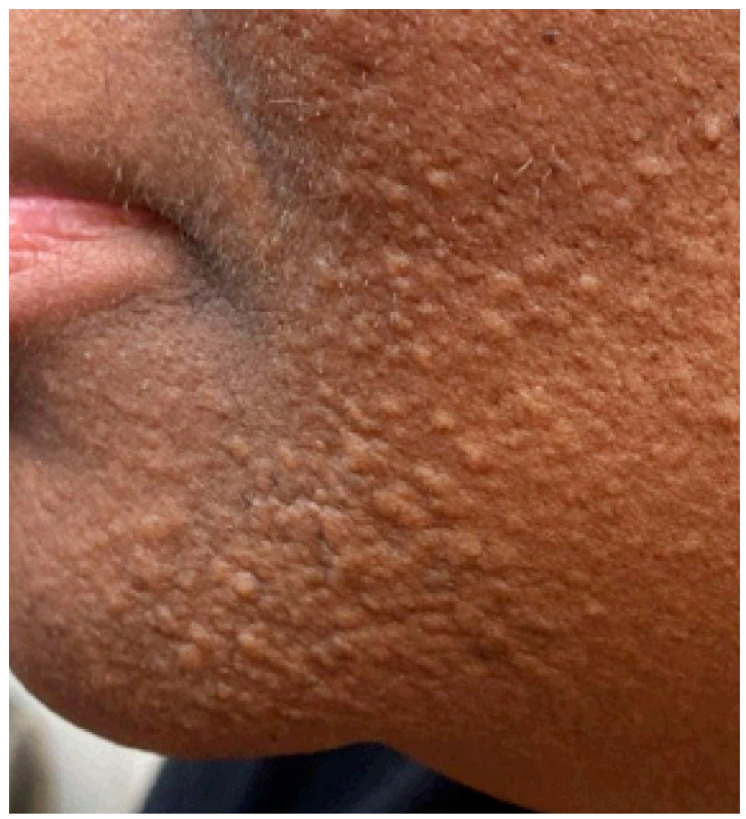
Papules coalescing into plaque over the medial left mandible.

**Figure A2:**
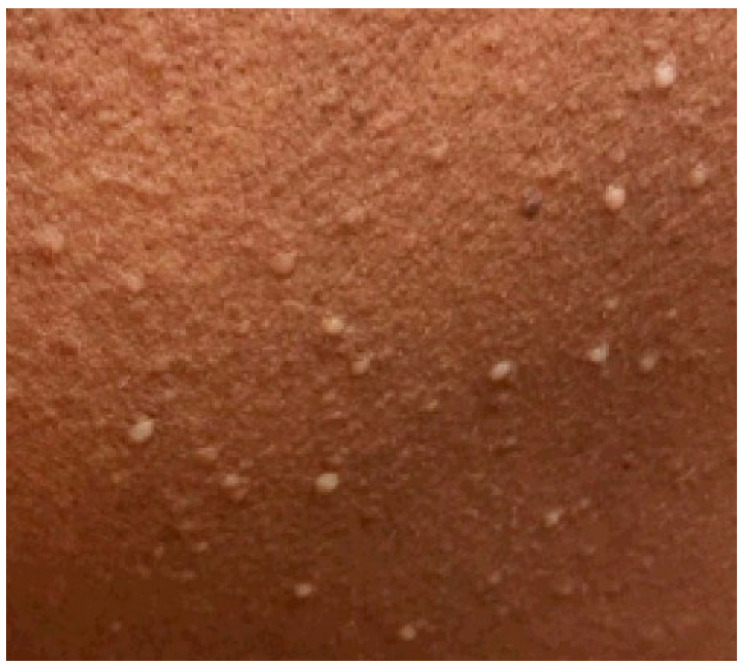
Waxy, white, flat-topped papules over the left buccal cheek.

## Data Availability

The original data presented in the study are included in the article, further inquiries can be directed to the corresponding author.

## Abbreviations

The following abbreviations are used in this manuscript:

IVIG	Intravenous Immunoglobulin
